# Changes in Resting Neural Connectivity during Propofol Sedation

**DOI:** 10.1371/journal.pone.0014224

**Published:** 2010-12-02

**Authors:** Emmanuel A. Stamatakis, Ram M. Adapa, Anthony R. Absalom, David K. Menon

**Affiliations:** Division of Anaesthesia, School of Clinical Medicine, University of Cambridge, Cambridge, United Kingdom; Indiana University, United States of America

## Abstract

**Background:**

The default mode network consists of a set of functionally connected brain regions (posterior cingulate, medial prefrontal cortex and bilateral parietal cortex) maximally active in functional imaging studies under “no task” conditions. It has been argued that the posterior cingulate is important in consciousness/awareness, but previous investigations of resting interactions between the posterior cingulate cortex and other brain regions during sedation and anesthesia have produced inconsistent results.

**Methodology/Principal Findings:**

We examined the connectivity of the posterior cingulate at different levels of consciousness. “No task” fMRI (BOLD) data were collected from healthy volunteers while awake and at low and moderate levels of sedation, induced by the anesthetic agent propofol. Our data show that connectivity of the posterior cingulate changes during sedation to include areas that are not traditionally considered to be part of the default mode network, such as the motor/somatosensory cortices, the anterior thalamic nuclei, and the reticular activating system.

**Conclusions/Significance:**

This neuroanatomical signature resembles that of non-REM sleep, and may be evidence for a system that reduces its discriminable states and switches into more stereotypic patterns of firing under sedation.

## Introduction

An all encompassing definition of consciousness is difficult. It is however easier to define level and content of consciousness with level being associated with arousal and content being associated with the content of subjective experience [1]. The neuroanatomical structures that underpin these processes are a topic of intense debate. Some experts support the view that consciousness depends on intact function of brain stem-thalamo-cortical arousal circuits [2] while others propose that consciousness is instantiated in fronto-parietal association cortices [3], [4], [5]. Classically, investigation of consciousness has involved comparing the neural response to tasks in the awake and sedated states. However, there is emerging evidence that the behavior of the resting brain under anesthesia and sedation may provide additional important insights in this area [6], [7].

Resting state fMRI involves measuring the blood oxygen level dependent (BOLD) signal for the study of sets of brain regions that function as coherent networks during rest. Raichle et al., 2001 [8] used quantitative positron emission tomography (PET) to demonstrate the existence of such a baseline state of human brain function that is remarkably uniform in the awake resting state and they used the term baseline default mode of brain function to describe activity in the regions involved. Greicius et al., [9] called this set of regions the default mode network (DMN). The DMN appears to be largely task independent and normally displays reduced activity during specific goal directed behavior [10]. This network primarily includes the posterior cingulate, the medial prefrontal cortex and bilateral parietal cortices [11]. The DMN was one of the first resting networks to be extensively studied, and early findings on the DMN still hold [12], in that the main areas identified to be part of this network remain the same despite a multitude of studies, there is increasing evidence that the DMN may comprise multiple dissociated components [13].

Studies of the DMN still dominate the field of resting state fMRI. However, other distinct collections of cortical/subcortical regions, that have been shown to co-activate during task-specific functional imaging, have also been shown to co-modulate their activity at rest. As early as 1995, Biswal et al. [14] demonstrated that fluctuations in activity during rest were coherent within the motor cortex. More recently, Beckmann et al. (2005) [15] expanded on this idea, using independent component analysis to demonstrate that distinct cortical/subcortical regions, shown to co-activate during task-specific functional imaging also co-modulate in activity at rest. The principle has been demonstrated many times over for the motor system [13], [16], and also for visual [17], [18], auditory [18], memory [19], [20] and attention systems [21]. The emerging inference is that distinct neuroanatomical systems that co-activate in response to stimuli also display levels of distinct organization at rest.

The functionality of most of these systems can be easily inferred. However, the role of the DMN is still under discussion. There is sufficient evidence to suggest that the DMN might be involved in both stimulus independent (inward) or stimulus dependent activity, and that it might not be as homogeneous as originally thought [22], [23], [24], [25]. Dahaene and Changeux (2005) [3] introduced the idea that the default mode network may be part of a global workspace [26], a set of cortical neurons whose role is to facilitate exchange of information between remote systems. More recently, He and Raichle (2009) [27] have championed the concept that fluctuations in the DMN may represent the imaging counterpart of Slow Cortical Potentials (SCPs), 1–4 Hz fluctuations in brain electrical activity that are argued to be important in consciousness. While plausible, these neuroanatomical models of consciousness require further support from experimental data. Studies that examine the behavior of the network under conditions of sedation or anesthesia may provide useful insights in this context.

Sedation is a pharmacologically induced, reversible state, characterized by dose-related impairment of cognitive functions, including attention and memory, but during which consciousness and awareness are maintained. Progressively increasing sedation results eventually in anesthesia which has been defined as “drug-induced loss of consciousness during which patients are not rousable, even by painful stimulation.” [28]. In contrast to sedation, conscious perception of environmental stimuli is ablated during anesthesia, which may be mediated by mechanisms distinct from those mediating sedation.

The few studies that have explored DMN connectivity under sedation/anesthesia (see Table S1) have shown little change between sedated/anesthetized and awake states [6], [7], a surprising finding, given the overt behavioral changes that occur with deepening sedation/anesthesia. The small changes reported are also somewhat contradictory, in that Greicius et al., [6] reported focal increases in connectivity in the sensory-motor network with sedation, while Martuzzi et al., [7] found no significant changes in connectivity in sensory cortices when comparing pre- and anesthetized conditions. This discordance requires explanation.

In order to investigate this issue, we used the anesthetic agent propofol, to modulate consciousness and to evaluate differences in interaction of remote neural networks during altered consciousness. Propofol is an agonist at the GABA receptor (subtype A) and it is through this agonist activity that propofol exerts its sedative and hypnotic effect. In addition, it has agonist activity at the glycine receptor and mild antagonistic activity at the neuronal acetylcholine, AMPA and NMDA receptors [29]. Anesthesia with propofol causes predictable changes in EEG patterns, some of which are not dissimilar to those found in sleep. Light surgical levels of propofol anesthesia cause a similar EEG pattern to that found in stage II sleep i.e. attenuation of alpha and beta frequencies, and an increase in power in the theta range. A defining EEG feature of stage II sleep is the presence of sleep spindles, and spindles of similar morphology are also found in the EEG during deep propofol anesthesia [30]. Moreover, neuronal pathways involved in arousal from sleep have been implicated in recovery from anesthetic-induced loss of consciousness [31].

Propofol was administered using a computer controlled intravenous infusion [32], [33] aiming to achieve three *target* plasma levels - no drug (awake), low sedation and moderate sedation. Propofol has been shown to significantly reduce regional cerebral blood flow (rCBF) in the posterior cingulate cortex (PCC), medial thalamus, and basal forebrain, areas commonly implicated in arousal mechanisms and information processing/integration [34]. It has been argued that the PCC is important for consciousness/awareness [35], and it is widely recognized that the PCC is also a major hub in the default mode network [8]. We hypothesized that any sedation induced activity changes in the PCC would also affect the default mode network. We therefore acquired resting state fMRI BOLD data to investigate functional connectivity changes in the default network under propofol sedation.

## Results

At a propofol plasma target concentration of 0.6 µg/ml (mean [SD] *measured* plasma concentration 0.27 [0.12] µg/ml) all volunteers displayed a lethargic response to their name spoken in a normal tone (corresponding with an Observer's Assessment of Alertness/Sedation Scale (OAA/S) score of 4, or a Ramsay score of 2). At a propofol plasma target concentration of 1.2 µg/ml (mean [SD] *measured* plasma concentration 0.67 [0.36] µg/ml), all volunteers were more deeply sedated and responded only after their name was called loudly (corresponding with an OAA/S score of 3, or a Ramsay score of 3). Measured plasma concentration levels were significantly different (p≤0.001) when comparing low to moderate sedation.

An analysis of motion parameters (see Table S2) obtained during the realignment stage of preprocessing showed no relationship between sedation depth and movement. We carried out three ANOVAs, none of which produced significant results (Translation in x F(2,47) = 0.08; p = 0.92, in y F(2,47)  = 0.34; p = 0.71, in z F(2,47)  = 0.84; p = 0.43. Rotation in x F(2,47) = 0.42; p = 0.66 in y F(2,47)  = 0.74; p = 0.48 in z F(2,47)  = 0.29; p = 0.75). Thus, the data we present here were not significantly influenced by movement during scanning.

**Figure 1:**
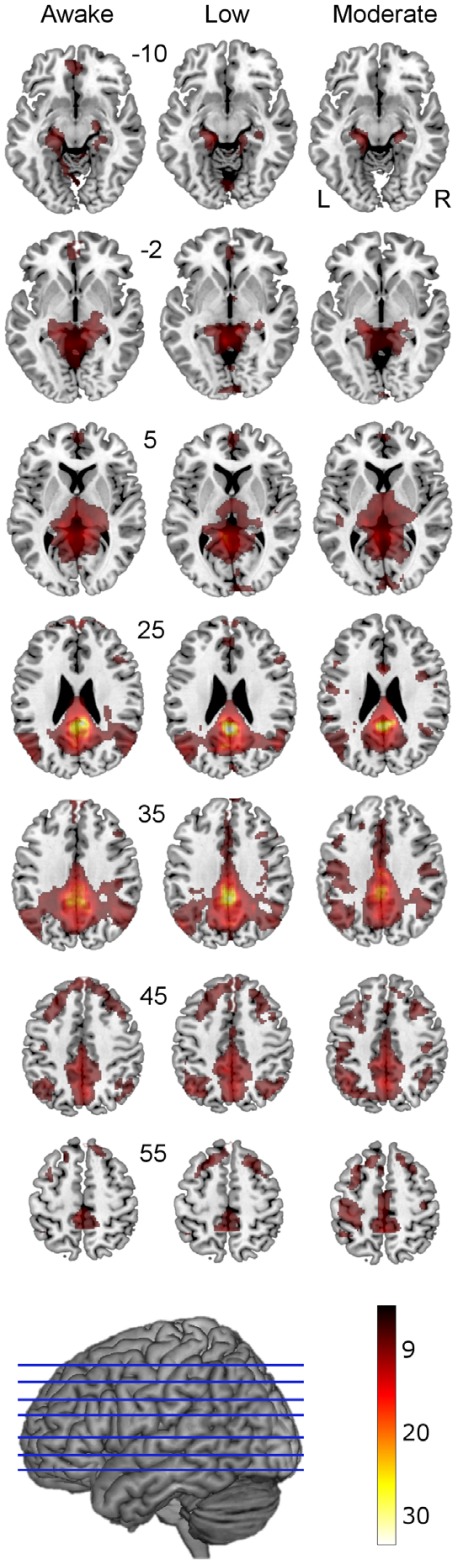
PCC connectivity at awake, low and moderate levels of sedation shown superimposed on a standard T1 weighted structural image. Talairach and Tournoux z coordinates are shown. The connectivity of the PCC appears to increase with sedation, especially in motor and somatosensory areas. We report clusters that survived a voxel threshold of p≤0.001 uncorrected and a random field cluster threshold of p≤0.05 corrected for the entire brain. The color bar indicates strength of connectivity (t score).

Following preprocessing, the imaging data was subjected to a group level one-sample t-test to find areas whose activity correlated with that of the PCC at the awake stage; in this manner we attempted to identify a resting network close to the DMN in our study population. The one-sample t-test revealed that PCC connectivity in awake subjects (Figure 1, Table 1) involved bilateral posterior cingulate cortices, precunei, lateral inferior parietal gyri (extending to angular gyri), superior medial frontal (extending to dorsal middle frontal) gyri, posterior hippocampi and parahippocampal gyri and thalami. These areas are concordant with previous studies utilizing multiple approaches to define the DMN [25].

**Table 1:** Significant connectivity peaks for the PCC connectivity at the awake stage.

Cluster p(cor)	Cluster size (vox)	Voxel (T)	Voxel (x y z)	Brain Region
<0.001	21515	35.87	6 −40 28	R Cingulate G BA 31
		35.55	−4 −48 20	L Post Cingulate G BA 30
		25.99	−4 −36 40	L Cingulate G BA 31
<0.001	3334	9.52	2 60 30	R Medial Frontal G BA 9
		9.05	−4 58 40	L Medial Frontal G BA 8

Cluster significance (p value) corrected for multiple comparisons is shown as well as the size of each cluster in voxels (voxel volume = 2 mm^3^) and the T score for the most significant voxel in the cluster. The x,y,z coordinates in Talairach and Tournoux space [74] for the most significant voxel are also shown as well as the name of the brain region that corresponds to the most significant voxel as named by Talairach and Tournoux [74].

**Figure 2:**
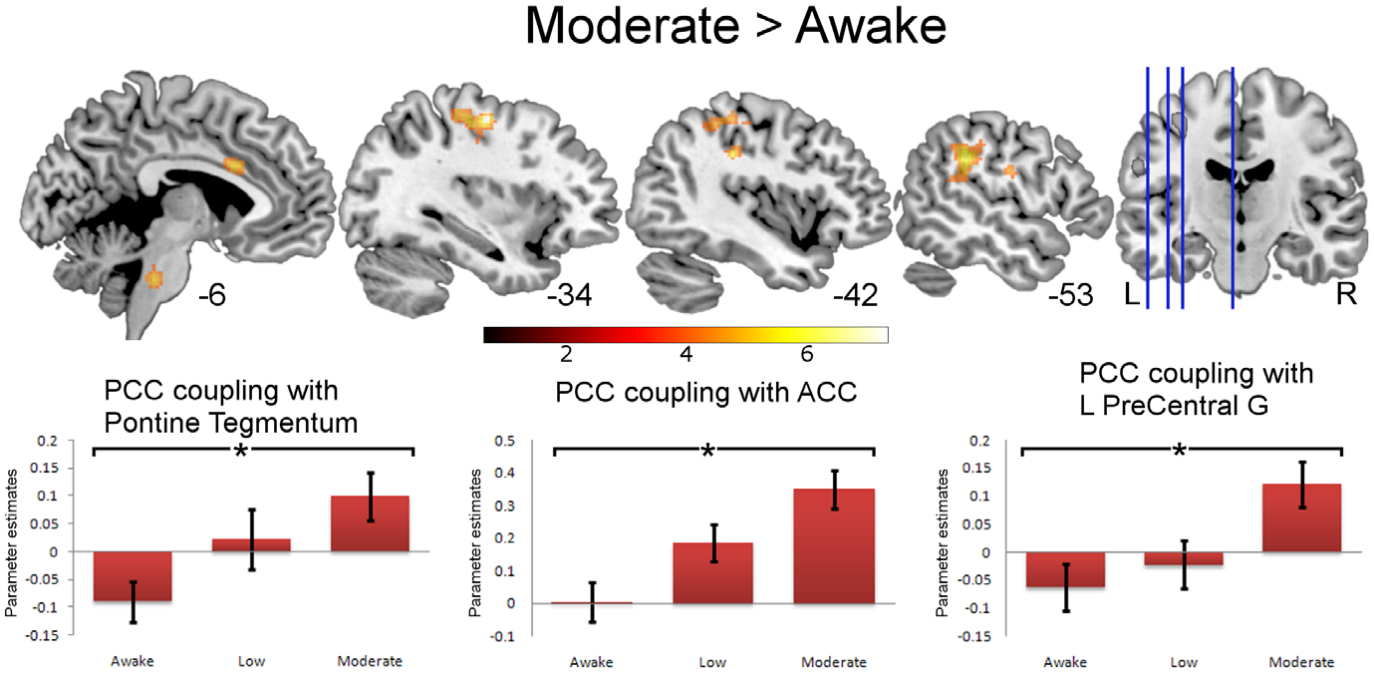
Significant PCC connectivity for Moderate>Awake sedation shown superimposed on a standard T1 weighted structural image. * in the plots denotes connectivity that survived a random field cluster threshold of p≤0.05 corrected for the entire brain (at voxel p≤0.001 uncorrected). Error bars show standard error. Talairach and Tournoux x coordinates shown underneath the T1 weighted brain slices. The color bar indicates strength of connectivity (t score).

During low and moderate sedation, group level one-sample t-tests suggested that PCC connectivity decreased in medial prefrontal areas (see Figure 1 z = −10, −2, 5 and 25). However, paired t-tests did not reveal statistically significant changes in connectivity from awake to moderate sedation in any area conventionally considered to be part of the DMN. Instead, we found statistically significant increases in connectivity between the PCC and the anterior cingulate cortex (ACC) (BA23, extending forward to the posterior extent of BA32), L supramarginal, L pre/postcentral gyri (motor/somatosensory cortex), and the pontine tegmentum (part of ascending reticular activating system-ARAS) in the brainstem (Figure 2, Table 2).

**Table 2:** Significant connectivity peaks for the contrast of moderate sedation vs. awake.

Cluster p(cor)	Cluster size (vox)	Voxel (T)	Voxel (x y z)	Brain Region
<0.001	307	7.55	−34 −12 54	L Precentral G BA 4
		6.10	−38 −30 56	L Inf Parietal Lobule BA 40
		5.40	−34 −22 52	L Precentral G BA 4
<0.001	327	7.08	6 −30 −36	R Pontine Tegmentum
		6.35	−12 −32 −40	L Pontine Tegmentum
		5.09	−2 −28 −36	L Pontine Tegmentum
<0.001	483	6.16	−56 −36 34	L Inf Parietal Lobule BA 40
		6.11	−44 −30 34	L Inf Parietal Lobule BA 40
		5.72	−60 −22 28	L Inf Parietal Lobule BA 40
0.002	215	5.88	−18 −54 −44	L Post Lobe Cerebellar Tonsil
		5.16	−26 −58 −42	L Post Lobe Cerebellar Tonsil
0.001	234	5.86	4 12 28	R Cingulate G BA 24
		5.40	−4 14 28	L Cingulate G BA 24
		5.26	12 22 24	R Ant Cingulate BA 32

Cluster significance (p value) corrected for multiple comparisons is shown as well as the size of each cluster in voxels (voxel volume = 2 mm^3^) and the T score for the most significant voxel in the cluster. The x,y,z coordinates in Talairach and Tournoux space [74] for the most significant voxel are also shown as well as the name of the brain region that corresponds to the most significant voxel as named by Talairach and Tournoux [74].

An inspection of Figure 2 reveals that the alterations in connectivity that we observed with sedation followed two different patterns. For the ACC, no connectivity with the PCC was observed at baseline, but sedation resulted in increases in positive correlation in activity in the two regions. However, for the precentral gyrus and pontine tegmentum, an initial significant negative correlation with the PCC in the awake state was transformed into a positive correlation in sedated subjects.

To estimate the level of regional BOLD signal fluctuation we calculated root mean squares (RMS) of percentage signal change for the PCC (where our DMN calculations originate) and the Left precentral gyrus (BA4) where we found the highest statistical peak for the comparison of moderate sedation vs. awake states (see Table S3 and Figure S1). We also plotted frequency distribution plots for these two regions (see Figure S2). In both regions there appears to be an increase in the overall power with moderate sedation - i.e. the overall amplitude of BOLD signal fluctuations appears to increase with sedation. This is supported by the RMS calculations shown in Table S3. Power appears to be increased in the BOLD fluctuations in two ranges at either end of the power spectrum: “waves” with period 10–60 seconds; and also in “waves” with period 2–3 seconds.

These differences in connectivity between awake and moderately sedated levels are based on predicted plasma propofol concentrations, calculated using a standard pharmacokinetic model for propofol [32], [33]. In order to account for the variability between predicted and observed propofol plasma levels, we carried out an additional whole brain regression analysis relating connectivity patterns to measured plasma propofol concentrations.

**Figure 3:**
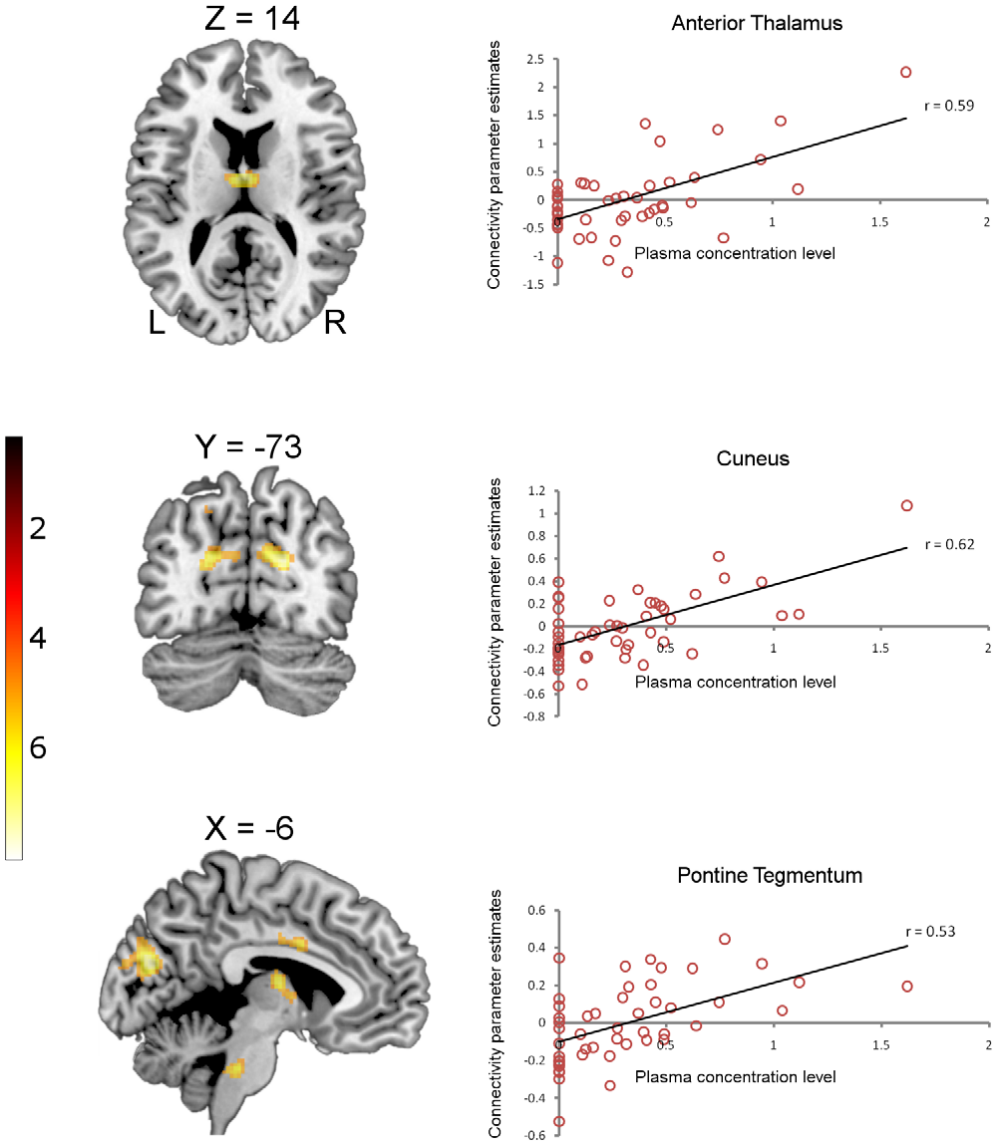
Increasing connectivity between the PCC and the thalamus (top panel), cuneus (middle panel) and the pontine tegmentum (bottom panel) in response to increasing propofol plasma concentrations (awake is modeled as zero). Correlations significant at p≤0.001: anterior thalamus r = 0.59, cuneus r = 0.62, pontine tegmentum r = 0.53. Talairach and Tournoux coordinates are shown above each T1 weighted brain slice. The color bar indicates strength of connectivity (t score).

The findings from this additional analysis were very similar to the comparison with predicted concentrations, described above. Measured plasma levels of propofol modulated connectivity between the PCC and the cunei, postcentral gyri (primary somatosensory cortex), precentral gyri (primary motor cortex) and premotor cortices; all bilaterally (Table 3). Although the sedation-associated increases in connectivity between the PCC and motor, premotor and somatosensory areas were bilateral, the clusters were substantially larger in the left hemisphere (see Table 3). Additionally, we found that plasma propofol concentration modulated connectivity between the PCC and the left putamen and bilateral thalami (mostly anterior nuclei, extending to bilateral medial dorsal nuclei). The connectivity clusters that we report here survived correction for multiple comparisons for the entire brain. We also found increased connectivity between the PCC and the ACC (survived correction for multiple comparisons for the entire cingulate gyrus) and in the pontine tegmentum (correction for multiple comparisons applied to the volume of the pons). We report these results which achieved significance with small volume corrections, both because of their recognized importance in neuroanatomical constructs of consciousness and anesthetic action, and because they were also significant in the initial paired t-test comparison (Figure 3).

**Table 3:** Significant connectivity peaks predicted by measured propofol plasma levels.

Cluster p(cor)	Cluster size (vox)	Voxel (T)	Voxel (x y z)	Brain Region
<0.001	1069	5.70	−54 −20 34	L Postcentral G BA 2
		5.14	−44 −14 34	L Precentral G BA 4
		4.85	−60 −32 30	L Inf Parietal Lobule BA 40
<0.001	1037	5.47	−14 −80 28	L Cuneus BA 18
		5.42	−8 −78 22	L Cuneus BA 18
		5.24	18 −72 20	R Cuneus BA 18
0.005	301	5.13	52 4 38	R Precentral G BA6
		4.23	52 −6 32	R Precentral G BA 6
		3.75	46 −18 32	R Postcentral G BA 3
0.010	260	5.02	2 −8 14	R Ant Nucleus Thalamus
		3.97	−18 4 8	L Putamen
		3.80	−10 −2 4	L Ant Nucleus Thalamus
0.007*	74	4.29	−6 −32 −34	L Pontine Tegmentum
		4.24	−8 −36 −36	L Pontine Tegmentum
0.015*	72	4.16	−6 4 32	L Cingulate G BA 24
		3.89	−4 −10 40	L Cingulate G BA 24
		3.73	−4 −8 36	L Cingulate G BA 24

Cluster significance (p value) corrected for multiple comparisons is shown as well as the size of each cluster in voxels (voxel volume = 2 mm^3^) and the T score for the most significant voxel in the cluster. The x,y,z coordinates in Talairach and Tournoux space [74] for the most significant voxel are also shown as well as the name of the brain region that corresponds to the most significant voxel, as named by Talairach and Tournoux [74].

*Significant after small volume correction.

## Discussion

We employed two different group level approaches to establish whether administration of the anesthetic agent propofol influences resting state connectivity of the PCC. The first was a direct comparison between awake and moderately sedated stages, and the second a regression analysis relating connectivity patterns to measured plasma propofol concentrations. These different approaches converged on similar findings. Increasing predicted or measured plasma propofol concentrations did not significantly modulate connectivity within the canonical default mode network, but were associated with significant increases in connectivity between the PCC and areas not conventionally thought to be part of the DMN: the motor/somatosensory cortices, the anterior thalamus, and the pontine tegmentum in the brainstem.

Before we ascribe a neural basis to these results, it is important that we consider whether they may be drug-induced changes in flow-metabolism coupling. Inhaled anesthetics can have independent effects on cerebrovascular physiology that confound interpretation of the BOLD response [36]. However, intravenous anesthetics such as propofol have been shown to have no major direct effects on cerebrovascular tone and to preserve flow-metabolism coupling [37], so the alterations in BOLD connectivity patterns we observed in our study are likely to reflect changes in neural activity. Further evidence for this comes from Veselis at al., 2005 [38] who measured rCBF responses during auditory stimulation (and quiet periods) at three levels of propofol effect: baseline, sedative, and hypnotic concentrations and concluded that sedative concentrations of propofol do not appear to influence the physiology associated with rCBF response to neural activity. The conclusion from these studies has to be that changes in rCBF are due to changes in neuronal activity because of the drug, and cannot be attributed to vascular changes. Consequently, any observed changes in connectivity patterns with sedative concentrations of propofol will represent effects on brain activity itself.

Given that the effects that we observed are likely to be neurally mediated, it is intriguing to contrast our results with previous PET and fMRI studies of anesthetic action that have used more classical approaches to image analysis (see Alkire et al, 2008 [39] for a concise review of these data). These studies suggest that graded propofol anesthesia reduces regional cerebral blood flow (rCBF) in the medial thalamus, posterior cingulate, basal forebrain, and in the occipitoparietal association cortices, areas commonly implicated in arousal mechanisms and information processing. In the context of these findings of reduced rCBF, our finding that connectivity increases with moderate sedation may seem somewhat counterintuitive.

However, it is important to distinguish between alterations in levels of activity in individual brain regions from changes in connectivity between regions. Our analysis of power frequency distribution of BOLD signal in the different regions of interest (see Figures S1 & S2 and Table S3) reveals that the changes in connectivity that we observed were associated with *increases* in power in distinct parts of the power spectrum. This argues against the possibility that our results were simply the consequence of incidental association in noisy data, and suggest that they are the consequence of biologically significant alterations in neural connectivity. The distinction between changes in absolute CBF [39] and changes in neural connectivity as manifest by concordant fluctuations in CBF is particularly relevant to data regarding resting state connectivity.

The number of published studies on the effects of anesthetic/sedative drugs on brain connectivity is still relatively small, and there is no consensus in this area. Differences between studies may arise from the fact that different anesthetic agents and/or methodologies have been employed in different studies (see Table S1). Peltier et al., 2005 [40] assessed changes in connectivity in motor areas during different levels of sevoflurane anesthesia, and reported a reduction in the number of significant voxels in connectivity maps, seeded from the primary motor cortex. Martuzzi et al., 2010 [7], who also used sevoflurane, analyzed their data using seed based connectivity analyses. They reported that, while anesthesia did not alter resting state connectivity significantly in the sensory cortices, it significantly changed connectivity in higher-order cognitive networks. They also found that anesthesia increased connectivity between a PCC seed and the superior temporal gyrus, but reduced connectivity between the seed and adjacent areas. Kiviniemi et al, 2005 [41], used midazolam sedation and reported increased temporal synchrony of the BOLD signal in auditory and visual cortices after sedation. Greicius et al., 2008 [6] also used midazolam to induce light sedation, and analyzed the data using independent component analysis (ICA) based on standard templates for different networks. This analysis showed increased connectivity in the sensory motor network (with focal increases in the mid-cingulate region), and decreased connectivity in the DMN (with focal decreases in the PCC).

The different levels of anesthesia/sedation may explain differences in the observed results; however the different mechanisms of the drugs used may also affect the results. For example sevoflurane also acts as a GABA-A agonist, but unlike propofol, it probably also acts at other receptors, such as TREK and TASK channels [42]. Although there are differences in the mechanism of action, the clinical states of sedation generated by the different anesthetic agents are quite similar. Additionally, midazolam has a fairly wide therapeutic range, compared with propofol, and in young and middle-aged patients, very large doses are required to cause loss of consciousness and loss of responses to pain.

Given the variable approaches adopted, it is unsurprising that there is no clear consensus regarding the impact of sedation and anesthesia on resting brain connectivity, and it has been, thus far, difficult to provide a rational mechanistic explanation for the changes observed. However, these apparently conflicting data may provide important insights into the issue. Several authors report sedation induced increases in connectivity between brain regions, although the regions involved vary depending on the analysis methodology (seed-based *vs.* ICA), the size of the seed employed, and the level of anesthesia/sedation studied. Specific networks where such increases in connectivity have been reported include the motor, somatosensory and visual networks. The increased connectivity between the PCC and non-DMN areas that we report add to this list, but is novel in that it describes connection between (rather than within) canonical resting state networks.

The connectivity increases we observe with sedation require a mechanistic explanation. The increases in sensorimotor connectivity have been, in previous publications, attributed to one of two explanations. The first of these is increased disinhibition [7], [34], [43], and a resulting increase in movement related artifact. However, both the Martuzzi et al., (2010) [7] analysis, and our data, show no significant differences in movement when comparing awake to sedated states. A second explanation, at least for increased connectivity between the thalamus and sensorimotor cortex [7], depends on an uneven modulation of cortico-thalamic feedback by anesthetic agents [44], with resulting prominence of the most robust connections. This hypothesis is supported by data proposing that propofol may act in such a manner [45]. Although we found alterations in connectivity involving somatosensory cortices, this explanation does not fully account for our results, because our connectivity analyses were not seeded from the thalamus or somatosensory cortices but from the PCC.

An alternative/complementary explanation for the increased connectivity we observed following the administration of propofol is based on the information integration theory of consciousness, which suggests that when the range of discriminable neuronal firing patterns shrinks, neural activity becomes less informative even though it may be more globally synchronized [46]. Evidence from human and animal literature indicates that both anesthesia and slow wave sleep (which are distinct phenomena associated with decreased arousal) force remote neural systems to switch to stereotypic responses that lack regional specificity, and that this can result in loss of information integration capacity (see [39] for an overview of action of anesthetic agents on brain activity). The increased connectivity patterns we observe in our data could be the BOLD expression of this underlying decrease in information integration produced by sedation.

Data from other studies support the inference that these increases in connectivity are associated with decreases in local activity. Previous studies have mapped the brain areas which show prominent reductions in neural activity associated with sedation and anesthesia, using imaging of both cerebral metabolism and absolute cerebral blood flow [47], [48], [34]. These areas of decreased activity map closely to the regions of increased connectivity that we demonstrate in our data, including involvement of the thalamus and the pontine tegmentum.

Thalamocortical loops have been associated with consciousness, and converging evidence from animal literature suggests that thalamic neurons switch out of the slow bursting mode and into fast gamma-band oscillations prior to awakening from sleep [49]. The increased connectivity we observe between the PCC and thalamus with increased sedation may be evidence for a change into a slow, more stereotypic firing pattern that conveys less information, as volunteers are increasingly sedated. The resulting loss of effective thalamocortical interactions provides one plausible cause of anesthetic-induced unresponsiveness [50]. It is also particularly intriguing that the increases in PCC connectivity to subcortical structures are not just limited to the thalamus, but also involves the pontine tegmentum, a key node in arousal networks [51]. The balance between brainstem mono-aminergic and cholinergic inputs is thought to be key in the development of REM sleep, with cholinergic inputs from the dorsal pons playing an important role in causing cortical arousal during REM sleep [49], [52], [53]. This, coupled with the deactivation of this region in previous studies of anesthesia [47], [48], suggests that propofol sedation not only suppresses processing of extrinsic stimuli [34], but may also obtund the intrinsic (de-afferented) cortical arousal associated with dreaming. Interestingly, dreaming is common just before return to consciousness after propofol anesthesia and when it occurs it is associated with REM type EEG patterns [54]. This interpretation of our data is concordant with previously reported commonalities in neural systems that subserve both sleep and anesthesia [55].

The location of the thalamic correlation in the anterior nuclei is significant for another reason: anterior thalamic nuclear lesions have been implicated in anterograde amnesia. Aggleton and Sahgal, 1993 [56] proposed that anterior thalamic dysfunction is an important factor in anterograde amnesia, since neurotoxic lesions in the anterior thalamic nuclei result in significant performance deficits in relevant neuropsychological tests. Additional evidence for the involvement of thalamic nuclei in memory function comes from research in Korsakoff's psychosis resulting from alcohol abuse. Harding et al., 2000 [57] showed that many of the cortical and subcortical regions involved in the encoding and retrieval of episodic memory are either unaffected (hippocampus) or damaged to the same extent (prefrontal cortex and basal forebrain) in both amnesic and non-amnesic alcoholics. However, neuronal loss in the anterior thalamic nuclei was found consistently only in patients with Korsakoff's psychosis. In the context of this evidence, the synchronization of the anterior thalamic nuclei with the PCC that we observe in our data may reflect a derecruitment of the anterior thalamus from normal mnemonic functions, and provide a neuroanatomical substrate for the amnesic action of propofol [58].

Our measured plasma propofol concentrations were significantly lower than values predicted by well established pharmacokinetic models. This is probably due to the fact that the infusion pumps that we used may have struggled to deliver the required infusion rates through the 8 m infusion lines used in the study. In the event, the absolute levels of propofol are less relevant to our analysis than the fact that we were able to achieve a range of plasma concentrations of the drug, which scaled concordantly with behavioral and imaging findings.

Given this concordance between propofol levels and fMRI findings, what might be the relationship between consciousness and the connectivity changes that we observe? Interpreted in the context of the information integration theory, which stipulates that consciousness increases in proportion to a system's repertoire of discriminable states [46], [59], our data suggest loss of independence in multiple discrete systems as they are de-recruited from their normal roles. We believe that the increased connectivity of non-DMN areas (such as the motor cortex, cuneus, pontine tegmentum, and thalamic nuclei) with the PCC represent examples of this phenomenon. If the DMN does indeed represent a global workspace [3], our data suggest that this global workspace, along with parts of other networks, and key brain regions involved in memory and arousal, is captured by the processes of sedation, and is no longer accessible for normal neural processing.

In this context, the changes in correlation between the PCC and the premotor cortex and pontine tegmentum that we show in Figure 2 are intriguing, with an inverse correlation in the awake state being transformed into a positive correlation in sedated subjects. The presence of anti-correlated BOLD fluctuations between task dependent regions and the PCC have been reported in the past [60]. However, the negative correlation that we observe between the pontine tegmentum and the PCC in the awake state is novel, and suggests that activation of the pontine reticular formation (which implies arousal) [61] is associated with reduction in resting activity in a key node of the DMN. One inference would be that arousal mechanisms in the brain stem deactivate the PCC in conscious resting subjects as part of normal wakefulness. However, the positive correlations that we observe between the PCC and pontine tegmentum in sedated subjects suggest that this normal feedback may be disrupted, as the pontine reticular formation, the DMN and parts of other neural systems are inactivated (but synchronized) by sedation.

In conclusion, we have shown, for the first time, evidence from an fMRI study that supports the idea of a system reducing its discriminable states and switching into more stereotypic patterns of firing under sedation. This process involves the DMN (as defined by activity that correlates with the PCC), components of the reticular activating system, the thalamus, and parts of the cortex. Our proposed explanation for these findings brings together observations from both sedation/anesthesia [6], [7], [40], [41] and sleep literature [42], [49], [53], [61], with a proposed key role for the DMN in consciousness and cognition. While this provides a plausible neuroanatomical substrate for the behavioral phenomenon of reduced consciousness with propofol, it remains unclear whether these changes are the consequence of a distributed function of the drug, or are heavily dependent on an effect of propofol at a strategic site (e.g, through potentiation of GABAergic outputs from the venterolateral preoptic nucleus [55]).

## Materials and Methods

### Participants

Having obtained local ethics permission from the Cambridgeshire 2 Regional ethics committee and written consent from participants we acquired resting state fMRI (150 volumes of BOLD data, 5 minutes, TR = 2 s) from a group of 16 adults, 19–52 years old (mean = 34.62, standard deviation = 9.05). During scanning we instructed volunteers to close their eyes and think about nothing in particular throughout the acquisition of the resting state BOLD data.

Volunteers were informed of the risks of propofol administration, such as loss of consciousness, respiratory and cardiovascular depression. They were also informed about more minor effects of propofol such as pain on injection, sedation and amnesia. In addition, standard information about intravenous cannulation, blood sampling and MRI scanning was provided.

Propofol was administered using a computer controlled intravenous infusion [32], [33] aiming to achieve three *target* plasma levels - no drug (awake), 0.6 µg/ml (low sedation) and 1.2 µg/ml (moderate sedation). At regular intervals between scans, depth of sedation was evaluated by assessing the responsiveness of volunteers to verbal instructions and formally recorded as OAA/S scores. In all volunteers two blood samples (2×1 ml) were taken at each sedation level for later measurement of plasma propofol concentrations with high performance liquid chromatography (HPLC).

The reasons why we chose to use fixed target concentrations, rather than titration of propofol dose to a sedation score, are as follows. All clinical sedation scales suffer from several weaknesses - they are highly subjective, and some of the commonly used scales, such as the Ramsay scale [62] suffer from significant inter-rater variability [63]. In addition most clinical sedation scales were designed for a different population than the one in our study. For example, the Ramsay scale, commonly used in sedation studies, was designed for agitated and anxious critically ill patients in an intensive care environment. The Observer's Assessment of Alertness/Sedation Scale (OAA/S) [64] is less well-known outside of the anesthesiology literature, but is more applicable to sedated healthy controls. Finally, sedation scores (including the OAA/S) correlate very poorly with objective EEG-based measures of sedation such as the Bispectral Index and state entropy [65], [66]. Nonetheless, in order to provide comparability with previous studies, we recorded data on sedation scores in all subjects.

During data collection there were always two trained anesthesiologists present, and observed the volunteer from the MRI control room and on a video link that showed the volunteer in the scanner. In addition, heart rate, electrocardiogram (ECG) and pulse oximetry were continuously monitored using an MR-compatible multiparameter monitor (Precess, InVivo Corp., Orlando, FL, USA). Non-invasive systemic blood pressure was measured intermittently during the study, but was suspended during scanning.

### Image acquisition

MRI data were acquired on a Siemens Trio 3T scanner (WBIC, Cambridge). Each functional BOLD volume consisted of 32 interleaved, descending, oblique axial slices, 3 mm thick with interslice gap of 0.75 mm and in-plane resolution of 3 mm, field of view  = 192×192 mm, repetition time = 2 s, acquisition time = 2 s, time echo = 30 ms, and flip angle 78. We also acquired T1-weighted structural images at 1 mm isotropic resolution in the sagittal plane, using an MPRAGE sequence with TR = 2250 ms, TI = 900 ms, TE = 2.99 ms and flip angle = 9°, for localization purposes.

### Data Analysis

fMRI (BOLD) images were pre-processed and modeled using SPM5 (Wellcome Institute of Imaging Neuroscience, London, UK) implemented in MATLAB version 6.5 (Mathworks, Natick, MA). The first six volumes were discarded to allow for MR signal equilibration. Pre-processing involved at first, slice-timing correction to correct differences in image acquisition time between slices. We then carried out within-subject realignment to account for head motion. The process involves realigning all images acquired during the acquisition of resting state fMRI to the mean image of the session using linear transformations. The realignment process produces a text file with the amount of translation (in x,y,z directions) and rotation (also in x,y,z directions) required for each functional image in order to bring it to the space of the mean image. The mean image produced from the realignment stage was then spatially normalized to an MNI template image, using a 12-parameter linear affine transformation (translation, rotation, zoom, and shear in x, y, and z directions) and a linear combination of three dimensional discrete cosine transform basis functions. The transformation parameters obtained from the spatial normalization of the mean to the standard space (MNI) were applied to every functional image acquired during the resting state scanning session. Finally, the spatially normalized images were smoothed with an isotropic 8-mm^3^ full-width half-maximum Gaussian kernel and high pass filtered at a cut off frequency of 0.008 Hz to remove any baseline drifts.

Motion parameters calculated during the realignment stage of preprocessing were subjected to statistical analysis to establish whether volunteers moved more during sedation. The assumption we tested was that light sedation may reduce inhibitory control and subsequently increase movement which may affect the imaging analysis differentially. We performed six single factor, three level (one for each awake, low and moderate sedation levels) ANOVAs for translation in x,y,z directions and rotation in x,y,z directions.

Following pre-processing, we used Marsbar [67] to extract seed time-series from the posterior cingulate cortex (PCC), white matter (WM) and CSF masks. We obtained a mean value per mask for each EPI image and defined PCC, WM and CSF masks anatomically using the WFU PickAtlas [68] (see Tzourio-Mazoyer et al., 2002 [69] for anatomical definition of PCC).

The most common approach to calculating DMN connectivity is to take a time-series extracted from the mean value of a sphere with its centre in the PCC or precuneus (PC). However, published literature includes a variety of candidate coordinates defining the centre of this sphere (e.g. Fox et al., 2005 [70] use −5, −49, 40 while Chang and Glover [71], 2010 use −6, −58, 28). This approach may produce variable results, depending on the candidate seed region selected. In order to provide maximum sensitivity, we adopted the approach described by Horovitz et al., 2009 [72], and used a mean value for the entire PCC. Further support for this approach comes from Buckner et al., 2008 [25] who suggest that the retrosplenial portion of the PCC is one of the core regions of the DMN.

We computed PCC connectivity for each subject using the time series from the PCC as the variable of interest in a linear regression model that also included the six movement parameters (calculated during realignment), global mean, global white matter and global CSF signals as variables of no interest [70]. We regressed out WM and CSF to remove any non-tissue specific confounding effects and to remove fluctuations unlikely to be involved in specific regional correlations [70].

Parameter estimates images were combined in group-level random effects analyses. We report clusters that survived a voxel threshold of p≤0.001 uncorrected and a random field cluster threshold of p≤0.05 corrected for the entire brain, unless otherwise stated.

To account for the variability between predicted and observed propofol plasma levels, we also carried out a regression analysis relating connectivity patterns to measured plasma propofol concentrations. We report results that survived a voxel threshold of p≤0.001 uncorrected and a random field cluster threshold of p≤0.05 corrected for the entire brain. We report two additional clusters (in the ACC and the pontine tegmentum) with small volume correction for multiple comparisons since we had *a priori* hypotheses for both of these.

We report MNI coordinates. However we converted these to Talairach space [73] before Talairach and Tournoux (1988) [74] nomenclature was assigned to peak activation voxels.

## Supporting Information

Table S1 fMRI studies that have investigated changes in connectivity during sedation.
(0.03 MB DOC)

Table S2 Average movement for each participant in mm and degrees as calculated by the realignment algorithm in SPM5. A-awake, L- low sedation, M-moderate sedation, Tx- average translation in x direction, Ty- average translation in y direction, Tz- average translation in z direction, Rx- average rotation in x direction, Ry- average rotation in y direction, Rz- average rotation in z direction.
(0.06 MB DOC)

Table S3 Percentage signal changes for the three different levels of sedation for the PCC (where our DMN calculations originate from) and the Left Precentral Gyrus (BA4) where we found the highest statistical peak for the comparison of moderate sedation vs. awake states. RMS- root mean square; SE- standard error.
(0.03 MB DOC)

Figure S1 Percentage signal changes for the three different levels of sedation for the PCC (where our DMN calculations originate from) and the Left Precentral Gyrus (BA4) where we found the highest statistical peak for the comparison of moderate sedation vs. awake states.
(0.10 MB TIF)

Figure S2 Frequency distribution plots for the PCC and the Left precentral gyrus. In both regions there appears to be an increase in the overall power with moderate sedation - i.e. the overall amplitude of BOLD signal fluctuations appear to be increasing. This is supported by the RMS calculations shown on Table S3 and Figure S1. Power appears to be increased in the BOLD fluctuations in two ranges at either end of the power spectrum.
(0.33 MB TIF)
