# Supplementary material for: Changes in Resting Neural Connectivity during Propofol Sedation
Source: PLoS One. 2010 Dec 2;5(12):e14224. doi: 10.1371/journal.pone.0014224 (PMC2996305; doi:10.1371/journal.pone.0014224)
Supplement: Table S1 — fMRI studies that have investigated changes in connectivity during sedation. (0.03 MB DOC) [file pone.0014224.s001.doc]

**Table S1.** fMRI studies that have investigated changes in connectivity during sedation.

| **Study** | **Drug used** | **Method of Analysis** | **Findings** |
| --- | --- | --- | --- |
| Peltier et al., 2005 | sevoflurane | Connectivity using a seed region in the motor cortex | Number of significant voxels for connectivity reduced during anesthesia |
| Kiviniemi et al., 2005 | midazolam | Power spectral and Signal synchrony analysis | BOLD fluctuation and synchrony increased in auditory and visual cortices during sedation |
| Greicius et al., 2008 | midazolam | Independent component analysis | Reduced connectivity with sedation in the DNM (PCC) but increased connectivity with sedation in sensory-motor network (mid-cingulate) |
| Martuzzi et al., 2010 | sevoflurane | Seed region based connectivity | No changes in DMN and sensory cortex connectivity with anesthesia but high-order cognitive regions (pain and memory) connectivity was significantly altered with anesthesia |
