# Supplementary material for: Changes in Resting Neural Connectivity during Propofol Sedation
Source: PLoS One. 2010 Dec 2;5(12):e14224. doi: 10.1371/journal.pone.0014224 (PMC2996305; doi:10.1371/journal.pone.0014224)
Supplement: Table S2 — Average movement for each participant in mm and degrees as calculated by the realignment algorithm in SPM5. A-awake, L- low sedation, M-moderate sedation, Tx- average translation in x direction, Ty- average translation in y direction, Tz- average translation in z direction, Rx- average rotation in x direction, Ry- average rotation in y direction, Rz- average rotation in z direction. (0.06 MB DOC) [file pone.0014224.s002.doc]

**Table S2.**Average movement for each participant in mm and degrees as calculated by the realignment algorithm in SPM5. A-awake, L- low sedation, M-moderate sedation, Tx- average translation in x direction, Ty- average translation in y direction, Tz- average translation in z direction, Rx- average rotation in x direction, Ry- average rotation in y direction, Rz- average rotation in z direction.

|  | **A_Tx** | **L_Tx** | **M_Tx** | **A_Ty** | **L_Ty** | **M_Ty** | **A_Tz** | **L_Tz** | **M_Tz** | **A_Rx** | **L_Rx** | **M_Rx** | **A_Ry** | **L_Ry** | **M_Ry** | **A_Rz** | **L_Rz** | **M_Rz** |
| --- | --- | --- | --- | --- | --- | --- | --- | --- | --- | --- | --- | --- | --- | --- | --- | --- | --- | --- |
| **subj1** | 0.023 | 0.525 | 0.683 | 0.069 | 0.059 | 0.046 | 0.116 | 0.310 | 0.345 | 0.004 | 0.004 | 0.002 | 0.000 | 0.000 | 0.002 | 0.000 | 0.005 | 0.009 |
| **subj2** | 0.008 | 0.071 | 0.113 | 0.079 | 0.099 | 0.068 | 0.030 | 0.517 | 0.351 | 0.001 | 0.013 | 0.001 | 0.000 | 0.000 | 0.007 | 0.001 | 0.001 | 0.001 |
| **subj3** | 0.489 | 0.007 | 0.002 | 0.081 | 0.100 | 0.077 | 1.854 | 0.906 | 0.702 | 0.008 | 0.012 | 0.003 | 0.011 | 0.003 | 0.001 | 0.005 | 0.001 | 0.000 |
| **subj4** | 0.124 | 0.269 | 0.043 | 0.028 | 0.055 | 0.249 | 0.398 | 0.305 | 0.295 | 0.003 | 0.001 | 0.031 | 0.002 | 0.001 | 0.006 | 0.000 | 0.005 | 0.001 |
| **subj5** | 0.019 | 0.011 | 0.019 | 0.101 | 0.087 | 0.202 | 0.235 | 0.172 | 0.093 | 0.005 | 0.000 | 0.012 | 0.000 | 0.001 | 0.001 | 0.000 | 0.000 | 0.004 |
| **Subj6** | 0.002 | 0.006 | 0.116 | 0.208 | 0.338 | 0.273 | 0.202 | 0.234 | 1.573 | 0.002 | 0.008 | 0.013 | 0.000 | 0.000 | 0.014 | 0.001 | 0.003 | 0.010 |
| **Subj7** | 0.505 | 0.564 | 0.184 | 0.281 | 0.266 | 0.236 | 0.462 | 0.430 | 0.467 | 0.015 | 0.012 | 0.002 | 0.001 | 0.001 | 0.001 | 0.004 | 0.009 | 0.002 |
| **Subj8** | 0.199 | 0.510 | 0.512 | 0.094 | 0.807 | 0.036 | 0.371 | 0.312 | 0.228 | 0.000 | 0.008 | 0.039 | 0.000 | 0.003 | 0.008 | 0.001 | 0.021 | 0.003 |
| **Subj9** | 0.450 | 0.410 | 0.183 | 0.135 | 0.030 | 0.125 | 0.104 | 0.690 | 0.482 | 0.001 | 0.019 | 0.012 | 0.001 | 0.001 | 0.004 | 0.005 | 0.007 | 0.053 |
| **subj10** | 0.004 | 0.065 | 0.007 | 0.010 | 0.173 | 0.036 | 0.598 | 0.686 | 0.441 | 0.007 | 0.013 | 0.003 | 0.002 | 0.000 | 0.003 | 0.000 | 0.005 | 0.003 |
| **subj11** | 0.055 | 0.044 | 0.044 | 0.093 | 0.075 | 0.031 | 0.491 | 0.185 | 0.134 | 0.002 | 0.000 | 0.000 | 0.000 | 0.000 | 0.000 | 0.000 | 0.002 | 0.000 |
| **subj12** | 0.011 | 0.261 | 0.016 | 0.219 | 0.044 | 0.150 | 0.120 | 0.120 | 0.326 | 0.004 | 0.033 | 0.039 | 0.003 | 0.015 | 0.005 | 0.002 | 0.003 | 0.004 |
| **subj13** | 0.000 | 0.437 | 0.541 | 0.268 | 0.418 | 0.277 | 0.437 | 0.122 | 0.766 | 0.003 | 0.008 | 0.005 | 0.005 | 0.004 | 0.004 | 0.006 | 0.003 | 0.005 |
| **subj14** | 0.061 | 0.236 | 0.274 | 0.198 | 0.403 | 0.545 | 0.920 | 0.624 | 0.206 | 0.002 | 0.015 | 0.017 | 0.001 | 0.005 | 0.007 | 0.001 | 0.006 | 0.029 |
| **subj15** | 0.213 | 0.257 | 0.142 | 0.010 | 0.104 | 0.175 | 0.103 | 0.065 | 0.017 | 0.000 | 0.001 | 0.004 | 0.003 | 0.005 | 0.005 | 0.004 | 0.001 | 0.001 |
| **subj16** | 0.213 | 0.257 | 0.142 | 0.010 | 0.104 | 0.175 | 0.103 | 0.065 | 0.017 | 0.000 | 0.001 | 0.004 | 0.003 | 0.005 | 0.005 | 0.004 | 0.001 | 0.001 |
