# Supplementary material for: Changes in Resting Neural Connectivity during Propofol Sedation
Source: PLoS One. 2010 Dec 2;5(12):e14224. doi: 10.1371/journal.pone.0014224 (PMC2996305; doi:10.1371/journal.pone.0014224)
Supplement: Table S3 — Percentage signal changes for the three different levels of sedation for the PCC (where our DMN calculations originate from) and the Left Precentral Gyrus (BA4) where we found the highest statistical peak for the comparison of moderate sedation vs. awake states. RMS- root mean square; SE- standard error. (0.03 MB DOC) [file pone.0014224.s003.doc]

**Table S3 and Figure S1.** Percentage signal changes for the three different levels of sedation for the PCC (where our DMN calculations originate from) and the Left Precentral Gyrus (BA4) where we found the highest statistical peak for the comparison of moderate sedation vs. awake states. RMS- root mean square; SE- standard error.

|  | **Awake-RMS of % signal change** | **SE** | **Low-RMS of % signal change** | **SE** | **Moderate-RMS of % signal change** | **SE** |
| --- | --- | --- | --- | --- | --- | --- |
| **PCC** | 0.308 | 0.018 | 0.319 | 0.023 | 0.453 | 0.046 |
| **LPRECENTRAL** | 0.219 | 0.014 | 0.223 | 0.011 | 0.371 | 0.043 |
