# Supplementary figures and images for: Changes in Resting Neural Connectivity during Propofol Sedation
Source: PLoS One. 2010 Dec 2;5(12):e14224. doi: 10.1371/journal.pone.0014224 (PMC2996305; doi:10.1371/journal.pone.0014224)

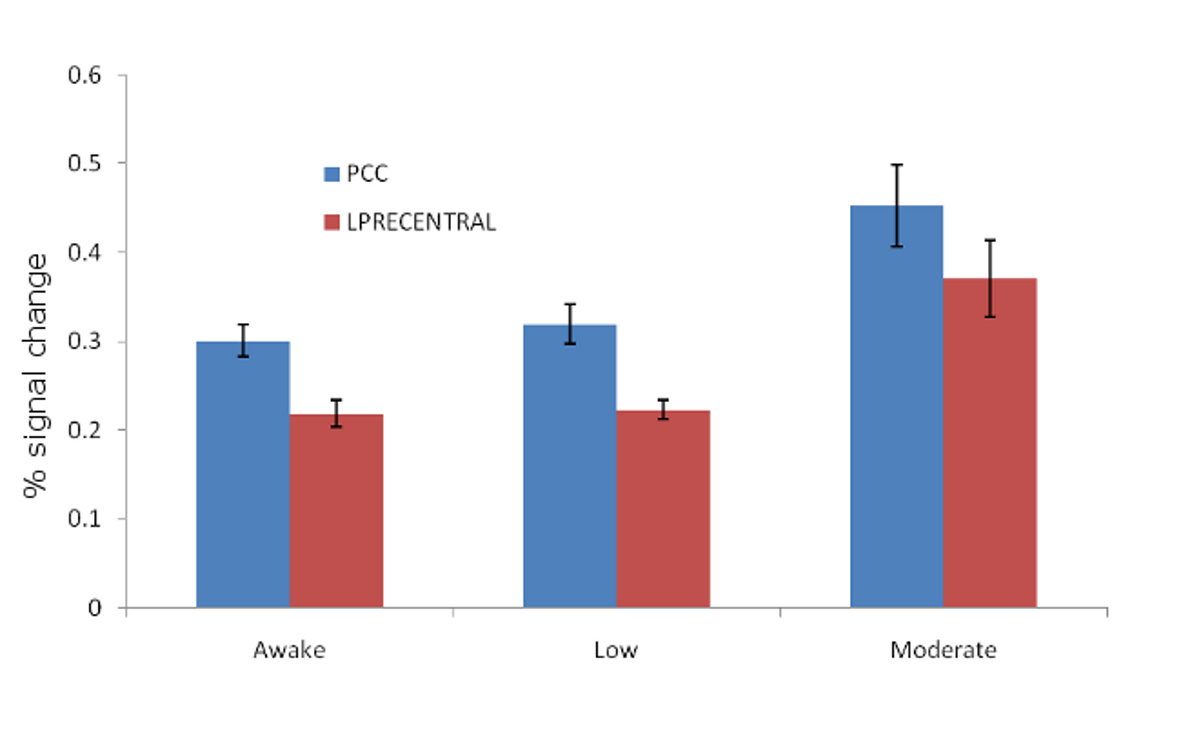

Supplement: Figure S1 — Percentage signal changes for the three different levels of sedation for the PCC (where our DMN calculations originate from) and the Left Precentral Gyrus (BA4) where we found the highest statistical peak for the comparison of moderate sedation vs. awake states. (0.10 MB TIF) [file pone.0014224.s004.tif]

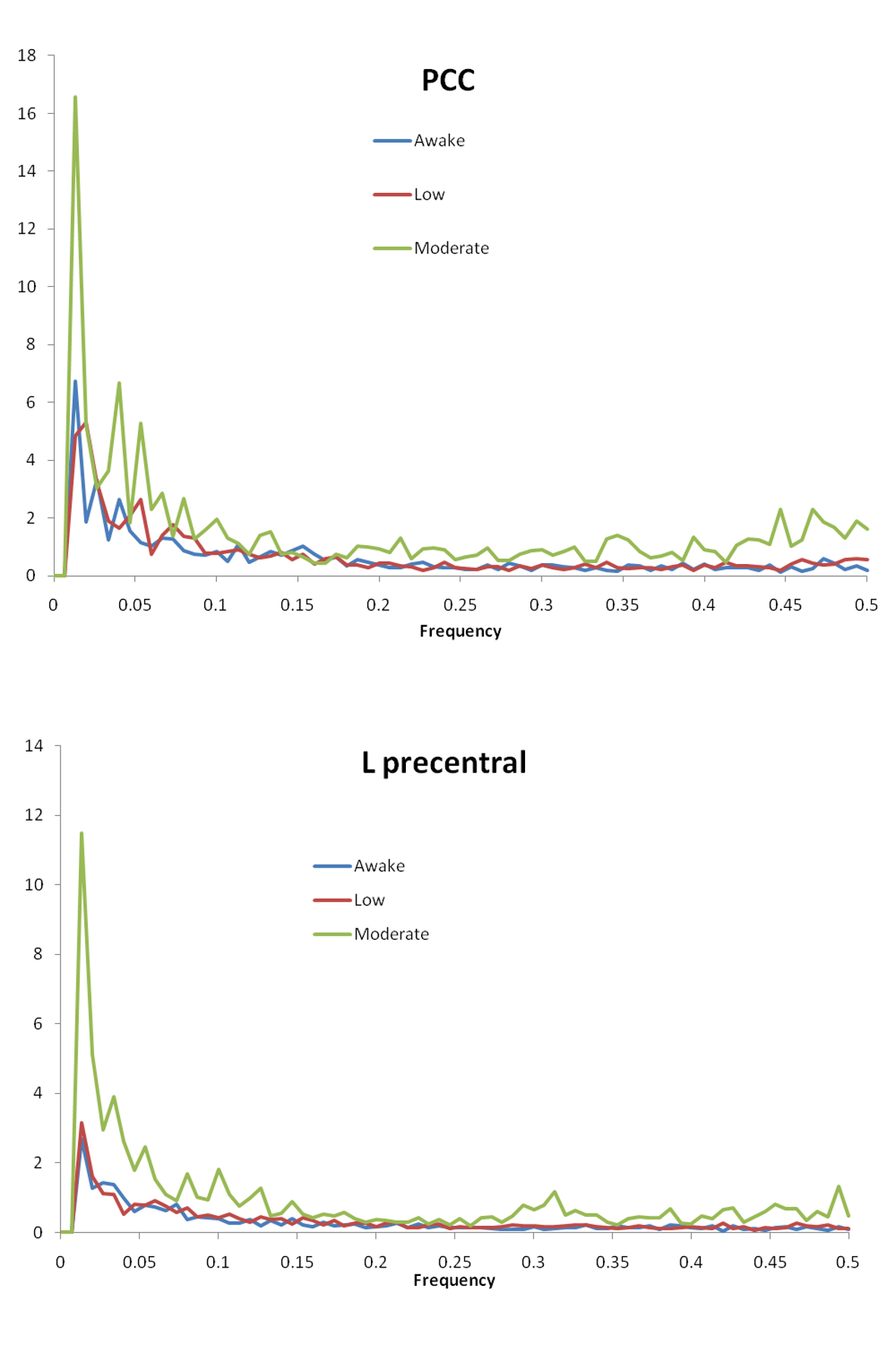

Supplement: Figure S2 — Frequency distribution plots for the PCC and the Left precentral gyrus. In both regions there appears to be an increase in the overall power with moderate sedation - i.e. the overall amplitude of BOLD signal fluctuations appear to be increasing. This is supported by the RMS calculations shown on Table S3 and Figure S1. Power appears to be increased in the BOLD fluctuations in two ranges at either end of the power spectrum. (0.33 MB TIF) [file pone.0014224.s005.tif]
